# Adsorption of Sc on the Surface of Kaolinite (001): A Density Functional Theory Study

**DOI:** 10.3390/ma16155349

**Published:** 2023-07-29

**Authors:** Zilong Zhao, Kaiyu Wang, Guoyuan Wu, Dengbang Jiang, Yaozhong Lan

**Affiliations:** 1 School of Materials and Energy, Yunnan University, Kunming 650091, China; zhaozilong@itc.ynu.edu.cn (Z.Z.); w13835318000@163.com (K.W.); wgy66@tom.com (G.W.); 2 Green Preparation Technology of Biobased Materials National & Local Joint Engineering Research Center, Yunnan Minzu University, Kunming 650500, China

**Keywords:** rare earth, scandium, kaolinite, density functional theory, adsorption

## Abstract

The adsorption behavior of Sc on the surface of kaolinite (001) was investigated using the density functional theory via the generalized gradient approximation plane-wave pseudopotential method. The highest coordination numbers of hydrated ${\mathrm{Sc}}^{3+}$, $\;\mathrm{Sc}{\left(\mathrm{OH}\right)}^{2+}$, and $\;\mathrm{Sc}{\left(\mathrm{OH}\right)}_{2\;}^{+}$ species are eight, six, and five, respectively. The adsorption model was based on $\mathrm{Sc}{\left(\mathrm{OH}\right)}_{2}{\left(H_{2}O\right)}_{5}^{+}$, which has the most stable ionic configuration in the liquid phase. According to the adsorption energy and bonding mechanism, the adsorption of Sc ionic species can be categorized into outer layer and inner layer adsorptions. We found that the hydrated Sc ions were mainly adsorbed on the outer layer of the kaolinite (001)Al-OH and (00−1)Si-O surfaces through hydrogen bonding while also being adsorbed on the inner layer of the deprotonated kaolinite (001)Al-OH surface through coordination bonding. The inner layer adsorption has three adsorption configurations, with the lying hydroxyl group (O_l_) position having the lowest adsorption energy (−653.32 KJ/mol). The adsorption energy for the inner layer is lower compared to the outer layer, while the extent of deprotonation is limited. This is because the deprotonation of the inner adsorption layer is energetically unfavorable. We speculate that Sc ions species predominantly adsorb onto the surface of kaolinite (001) in an outer layer configuration.

## 1. Introduction

In recent years, rare earth elements have been highly regarded due to their distinctive physical and chemical properties [1,2]. Among the most crucial strategic metals, they find extensive applications across numerous high-tech sectors [3,4,5,6]. According to their formation process, rare earth ores are primarily categorized into mineral and weathered types. Mineral-type rare earth ores serve as the primary source of light rare earth elements, typically represented by bastnasite and monazite [7]. On the other hand, weathered rare earth ores are medium in medium and heavy rare earth elements, with a notable example being weathered crust elution-deposited rare earth ore, also referred to as ion-adsorption-type rare earth ore [8]. Despite its low grade (0.05–0.2 wt.% rare earth oxides), weathered rare earth ore holds an 80% share in the global supply of medium and heavy rare earth elements [9]. 

Scandium (Sc) is a rare and expensive metal widely used in various industries such as electronics, optics, automotive, aerospace, transportation, and the production of advanced materials [10,11]. It falls into the heavy rare earths group due to its similar physical and chemical properties to the rare earth elements and its frequent occurrence in symbiosis with yttrium and lanthanides [12]. Rare earth elements primarily adsorb onto clay minerals in hydrated or hydroxy-hydrated forms [8]. Many scholars have investigated the coordination states of hydrated or hydroxy-hydrated rare earth ions in solution. Qiu et al. [13] employed a density functional theory to study the coordination states and bonding mechanisms of ${\mathrm{La}}^{3+}$ in aqueous environments and found that ${\mathrm{La}}^{3+}$ tends to coordinate with more water molecules. $\mathrm{La}{\left(H_{2}O\right)}_{10}^{3+}$ was found to be the most stable structure for La ions in low pH solutions, whereas $\mathrm{La}\left(\mathrm{OH}\right){\left(H_{2}O\right)}_{8}^{2+}$ was deemed more plausible in high pH solutions. Rudolph et al. [14] investigated the hydration of lanthanide(III) aqua ions in aqueous solutions and observed that light rare earth ions formed nona-hydrates while heavy rare earth ions formed octa-hydrates.

Typical ion-adsorption type rare earth ores include kaolinite, montmorillonite and illite, and mineralogical analyses have indicated that kaolinite is the main mineral of this type [15]. Kaolinite (${\mathrm{Al}}_{2}O_{3}\cdot 2{\mathrm{SiO}}_{2}\cdot 2H_{2}O$) is a 1:1 layered silicate characterized by the alternation of silicon–oxygen tetrahedra and aluminum–oxygen octahedra stacked along the c-axis [16]. Kaolinite layers are held together by interlayer hydrogen bonding, which restricts the diffusion of water molecules into the interlayer space. Therefore, kaolinite has a low coefficient of expansion and is prone to crack along the (001) plane [17,18,19]. As rare earth species in ion-adsorption-type rare earth ores are mainly adsorbed as ions on the surface of clay minerals, their separation can be enabled through ion exchange in electrolyte solutions (e.g., NaCl, ${\mathrm{NH}}_{4}\mathrm{Cl}$, or ${\left({\mathrm{NH}}_{4}\right)}_{2}{\mathrm{SO}}_{4}\;$ [20,21]), without the need for conventional mineral processing methods such as flotation or magnetic separation [22,23]. The current leaching process for ion-adsorbed-type rare earth ores mainly uses ${\left({\mathrm{NH}}_{4}\right)}_{2}{\mathrm{SO}}_{4}$ as the leaching agent and ${\mathrm{NH}}_{4}{\mathrm{HCO}}_{3}$ as the precipitant in the in situ leaching process. The in situ leaching process is less expensive and causes limited environmental damage than pool and heap leaching processes but suffers from various disadvantages such as poor leaching efficiency, ammonia and nitrogen pollutant emission, and difficulty in effectively recovering rare earth ions [24]. These issues result in a large amount of rare earths remaining in the waste residue, requiring secondary or tertiary leaching to recover the rare earth resources. Various factors contribute to the presence of residual rare earths in the waste residue, including blind spots in the leaching process, capillary phenomena, incomplete weathering, and desorption phenomena [25]. To address these problems, it is necessary to study the adsorption mechanism of rare earths at the molecular level in order to gain a deeper understanding of the adsorption mechanism and surface interactions. This will provide guidance for improving the efficiency and selectivity of metallurgical residue recovery and contribute to the development of new environmentally friendly and efficient leaching agents.

With the development of quantum chemical calculations, density functional theory (DFT) has found extensive applications in the field of mineral processing [26,27,28]. In recent years, this method has also been widely employed to investigate the adsorption of metal ions on the surfaces of various clay minerals, including kaolinite. Chen et al. [29] conducted density functional theory (DFT) calculations to investigate the adsorption of ${\mathrm{Hg}}^{2+}$ on the surface of kaolinite (001). The findings revealed that ${\mathrm{Hg}}^{2+}$ exhibits a maximum hydration coordination number of six in the liquid phase; they also determined its optimal adsorption site and bonding mechanism on the surface of kaolinite. Qiu et al. [30] conducted a study on the adsorption mechanism of $\mathrm{Lu}{\left(\mathrm{OH}\right)}_{2\;}^{+}$ on the surface of kaolinite. Their findings indicated that hydrated $\mathrm{Lu}{\left(\mathrm{OH}\right)}_{2\;}^{+}$ was more stable than hydrated ${\mathrm{Lu}}^{3+}$. Moreover, they reported that the adsorption energy of hydrated $\mathrm{Lu}{\left(\mathrm{OH}\right)}_{2\;}^{+}$ was higher for inner layer adsorption than for outer layer adsorption and that $\mathrm{Lu}{\left(\mathrm{OH}\right)}_{2\;}^{+}$ was preferentially adsorbed on the deprotonated Al-OH surface. These results suggest that the primary adsorption mechanism of rare earth ions on kaolinite involves outer layer adsorption, whereas the inner layer adsorption mechanism may dominate in high pH environments. Peng et al. [31] investigated the adsorption behavior of $Y{\left(\mathrm{OH}\right)}_{3-n\;}^{n+}$ (n = 1–3) on kaolinite surfaces at different degrees of deprotonation using DFT calculations. They found that the deprotonation reaction of the Al-OH surface was energetically unfavorable, with an energy of 145.5 KJ/mol. Furthermore, they observed that the surface activity and adsorption energy of rare earth ions increased as the degree of deprotonation of the adsorbed species on the kaolinite (001) surface increased. Consequently, the adsorption energies of $Y{\left(\mathrm{OH}\right)}_{3-n\;}^{n+}$ on kaolinite (001) and (00−1) surfaces gradually decreased with increasing degree of hydrolysis of $Y^{3+}$ ions. Yan et al. [32] conducted DFT calculations to investigate the adsorption of $\mathrm{Lu}{\left(\mathrm{OH}\right)}^{2+}$ and $\mathrm{Al}{\left(\mathrm{OH}\right)}^{2+}$ on kaolinite (001) and (00−1) surfaces. Their findings revealed that the coordination numbers of these two hydrated rare earth ions are seven and five, respectively. Additionally, they observed that both hydrated ions could be adsorbed on kaolinite Al-OH and Si-O surfaces through hydrogen bonding and on the deprotonated Al-OH surface through coordination bonding. They further noted that hydrated $\mathrm{Al}{\left(\mathrm{OH}\right)}^{2+}$ ions exhibit easier adsorption on kaolinite surfaces compared to hydrated $\mathrm{Lu}{\left(\mathrm{OH}\right)}^{2+}$, primarily due to their smaller radius and lower coordination numbers.

In this study, we investigated the hydration structures of ${\mathrm{Sc}}^{3+}$,$\;\mathrm{Sc}{\left(\mathrm{OH}\right)}^{2+}$, and $\;\mathrm{Sc}{\left(\mathrm{OH}\right)}_{2\;}^{+}$ in a liquid-phase environment via DFT calculations. In addition, the optimal hydrated configuration of $\mathrm{Sc}{\left(\mathrm{OH}\right)}_{2}{\left(H_{2}O\right)}_{5}^{+}$ in a liquid-phase environment was investigated for outer layer adsorption on the (001)Al-OH and (00−1)Si-O surfaces of kaolinite, as well as its mono-dentate inner layer adsorption on the deprotonated (001)Al-OH surface. Furthermore, the partial density of states (PDOS) and Mulliken charge population were also analyzed.

## 2. Theoretical Methods and Models

### 2.1. Calculation Methods and Parameters

DFT calculations were carried out based on the plane wave pseudopotential implemented using the Cambridge Sequential Total Energy Package (CASTEP) 19.1 software package [33,34,35,36]. The Perdew–Burke–Ernzerhof (PBE) functional in the generalized gradient approximation allows a more accurate description of the hydrogen bonding system consisting of water and has been used to analyze the exchange-correlation potential [37]. The ultrasoft pseudopotential (USP) was utilized to model the interaction between the ionic core and valence electron [38], and the Grimme method in DFT-D dispersion correction was employed to describe systemic weak interactions [39]. The plane-wave cut-off energy was set at 480 eV, and the model was geometrically optimized using the Broyden–Fletcher–Goldfarb–Shanno (BFGS) algorithm [40]. The convergence tolerance values were set as follows: maximum atomic force, 0.03 eV/Å; maximum atomic displacement, 0.001 Å; energy, 1.0 × 10^−5^ eV/atom; and maximum stress, 0.05 GPa. The K-point grids of Brillouin zone integrations used for the kaolinite bulk phase and surface are (3 × 2 × 2) and (3 × 2 × 1), respectively. The SCF converged to an accuracy of 2.0 × 10^−6^. The dipole moment correction was taken into account in the optimization and property evaluation of the surface.

### 2.2. Construction of Computational Models

**Figure 1:**
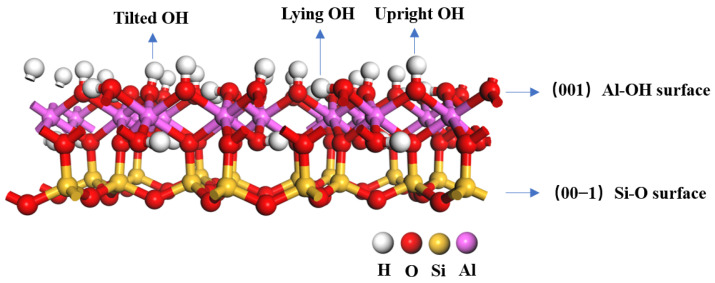
Structure of the kaolinite (001) surface.

The chemical formula of kaolinite is ${\mathrm{Al}}_{4}{\mathrm{Si}}_{4}{\left(\mathrm{OH}\right)}_{8}$, and we used the initial input cell structure of kaolinite with the space group C1, which was determined through low-temperature (1.5 K) neutron powder diffraction by Bish [41] in the USA [42,43]. The geometry of bulk kaolinite was optimized using the following lattice constants: a = 5.19 Å, b = 8.98 Å, c = 7.35 Å, α = 91.48°, β = 105.01°, and γ = 89.91°, within 2% error of experimentally measured cell parameters. We chose the (001) cleavage plane of kaolinite as the adsorption surface and set the vacuum layer thickness as 15 Å. The kaolinite surface was subjected to a 2 × 2 × 1 supercell. To optimize the two surfaces, the bottom three layers of atoms are fixed, and only the top three layers are relaxed. The optimized kaolinite surface is shown in Figure 1. The kaolinite (001) surface exposes the Al-OH layer, while the kaolinite (00−1) surface exposes the Si-O layer. After optimization, the Al-OH surface exhibits three distinct types of hydroxyl groups: tilted hydroxyl groups (O_t_H), lying hydroxyl groups (O_l_H), and upright hydroxyl groups (O_u_H) [44].

Due to the uncertain number of coordinated water molecules, models of all possible hydration and hydroxyl hydration configurations of ${\mathrm{Sc}}^{3+}$, viz., ${\left[\mathrm{Sc}{\left(\mathrm{OH}\right)}_{m}{\left(H_{2}O\right)}_{n}\right]}^{\left(3-m\right)+}$ (0 ≤ m ≤ 2, 1 ≤ n ≤ 10) were constructed and optimized to determine the most stable ionic configuration for adsorption analysis. The binding energy between ${\mathrm{Sc}}^{3+}$ and the $H_{2}O$ molecule is calculated as (1):

(1) $$\begin{array}{c} {\mathrm{Sc}}^{3+}+m{\left(\mathrm{OH}\right)}^{-}+nH_{2}O\to \mathrm{Sc}{\left(\mathrm{OH}\right)}_{m}{\left(H_{2}O\right)}_{n}^{\left(3-m\right)+}\\ E_{\mathrm{binding}}=E_{\mathrm{Sc}/H}-E_{\mathrm{Sc}}-{\mathrm{mE}}_{\mathrm{OH}}-{\mathrm{nE}}_{H_{2}O}{}_{\;} \end{array}$$

where E_binding_ represents the binding energy of the Sc ion hydrate, $E_{\mathrm{Sc}/H}$ denotes the total energy of the Sc ion hydrate, $E_{\mathrm{Sc}}$ refers to the energy of the Sc ion, m and n indicate the number of $H_{2}O$ and -OH species in the hydrate, and $E_{\mathrm{OH}}$ and $E_{H_{2}O\;}$ represent the energies of the -OH species and $H_{2}O$ molecules, respectively.

According to the different adsorption mechanisms of rare earth ions on kaolinite surfaces, rare earth adsorption can be categorized into outer layer and inner layer adsorption types [45]. The outer layer adsorption is due to physical adsorption, and the rare earth hydrates are bound to the kaolinite surface via hydrogen bonds, and the bonding force is weak. The inner layer adsorption is due to chemisorption, involving the formation of coordination bonds between rare earth hydrates and oxygen atoms on the deprotonated kaolinite surface, resulting in a strong bonding force [46]. In this study, rare earth hydrates were placed directly over the central Al atom on the surface of (001)Al-OH and over the central silicon ring on the surface of (00−1)Si-O as initial outer adsorption structures. The inner layer adsorption occurs exclusively on the (001) Al-OH surface, where a water molecule of the ${\mathrm{Sc}}^{3+}$ hydrate with a saturated coordination structure is removed and placed directly above deprotonated oxygen atoms with three different configurations (O_t_H, O_l_H, and O_u_H) around the Al atom at the center of the (001) Al-OH surface as the initial inner layer adsorption structure. The adsorption energy of the ${\mathrm{Sc}}^{3+}$ hydrate adsorbed on the outer and inner layers of the kaolinite surface are calculated as (2) and (3), respectively:

(2) $$\begin{array}{c} S\equiv \mathrm{Al}{\left(\mathrm{OH}\right)}_{3}+{\left[\mathrm{Sc}{\left(\mathrm{OH}\right)}_{2}{\left(H_{2}O\right)}_{n}\right]}^{+}\to \\ {\left[S\equiv \mathrm{Al}{\left(\mathrm{OH}\right)}_{3}\mathrm{Sc}{\left(\mathrm{OH}\right)}_{2}{\left(H_{2}O\right)}_{m}\right]}^{+}+\left(n-m\right)H_{2}O\\ E_{\mathrm{ads}}=E_{\mathrm{Sc}/S}-E_{\mathrm{Sc}}-E_{S} \end{array}$$

(3) $$\begin{array}{c} S\equiv \mathrm{Al}{\left(\mathrm{OH}\right)}_{3}+{\left[\mathrm{Sc}{\left(\mathrm{OH}\right)}_{2}{\left(H_{2}O\right)}_{n}\right]}^{+}+{\mathrm{OH}}^{-}\to \\ \left[S\equiv \mathrm{Al}{\left(\mathrm{OH}\right)}_{2}\left(O\right)\mathrm{Sc}{\left(\mathrm{OH}\right)}_{2}{\left(H_{2}O\right)}_{m}\right]+\left(n-m+1\right)H_{2}O\\ E_{\mathrm{ads}}=E_{\mathrm{Sc}/S}-E_{s}-E_{\mathrm{Sc}}+\left(n-m+1\right)E_{H_{2}O}-E_{{\mathrm{OH}}^{-}} \end{array}$$

where $S\equiv \mathrm{Al}{\left(\mathrm{OH}\right)}_{3}$ denotes the surface of kaolinite (001), n and m are the numbers of aqueous ligands of Sc ion hydrate before and after adsorption.${\;E}_{\mathrm{ads}}$ represents the adsorption energy of the system, $E_{\mathrm{Sc}/S}$ denotes the total energy of the system after adsorption, $E_{s}$ is the total energy of the kaolinite (001) surface, $E_{\mathrm{Sc}}$ is the energy of the Sc ion hydrate and, ${\;E}_{H_{2}O}$ is the energy of the water molecule. A negative value of the adsorption energy ($E_{\mathrm{ads}}$) indicates an exothermic reaction and a larger negative value signifies greater adsorption stability.

## 3. Results and Discussion

### 3.1. Geometric Configuration of ${\left[Sc{\left(OH\right)}_{m}{\left(H_{2}O\right)}_{n}\right]}^{\left(3-m\right)+}$

**Figure 2:**
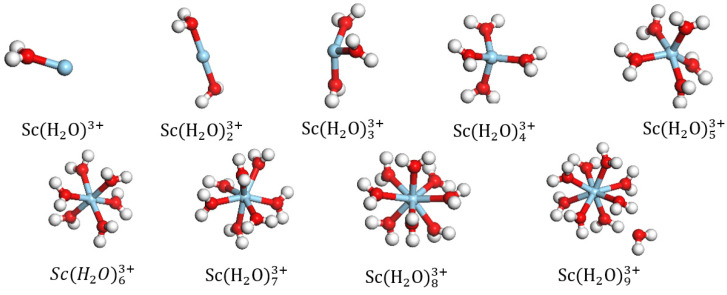
Equilibrium geometries of $\mathrm{Sc}{\left(H_{2}O\right)}_{1-9}^{3+}$ (blue ball represents Sc).

The equilibrium geometry of the DFT-optimized $\mathrm{Sc}{\left(H_{2}O\right)}_{1-9}^{3+}$ structures are shown in Figure 2. ${\mathrm{Sc}}^{3+}$ forms a coordination bond with the O atom of the $H_{2}O$ molecule. When the number of coordinated $H_{2}O$ was greater than eight, one of the $H_{2}O$ ligands in $\mathrm{Sc}{\left(H_{2}O\right)}_{9}^{3+}$ broke away from the first hydration layer of ${\mathrm{Sc}}^{3+}$ and became conformationally unstable; this observation suggests that ${\mathrm{Sc}}^{3+}$ can have a maximum coordination number of eight. The equilibrium geometrical parameters and binding energies of $\mathrm{Sc}{\left(H_{2}O\right)}_{1-9}^{3+}$ are presented in Table 1. As the number of aqueous ligands increases, the steric hindrance around ${\mathrm{Sc}}^{3+}$ increases, and the average bond length, R(Sc-O_w_)_avg_ between ${\mathrm{Sc}}^{3+}$ and O_w_ in aqueous ligands increases. ${\mathrm{Sc}}^{3+}$ gains electrons from the coordinated water molecules, and its charge tends to decrease as a result, as with the hydrate of the La ion [13]. As more water molecules are included, the binding energy of the rare earth hydrates decreases. A lower binding energy indicates a higher stability of the system. The adsorption of hydrated ${\mathrm{Sc}}^{3+}$ exhibits the most stable configuration with $\mathrm{Sc}{\left(H_{2}O\right)}_{8}^{3+}$, with a binding energy of −2629.44 KJ/mol. 

**Figure 3:**
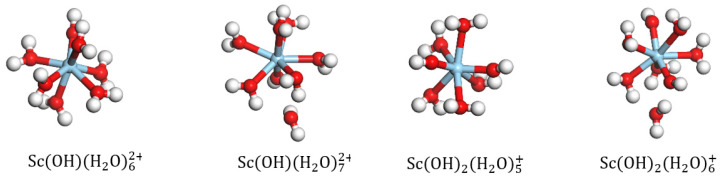
Equilibrium geometries of $\mathrm{Sc}\left(\mathrm{OH}\right){\left(H_{2}O\right)}_{n}^{2+}$ and $\mathrm{Sc}{\left(\mathrm{OH}\right)}_{2}{\left(H_{2}O\right)}_{n}^{+}$.

The water molecule in $\mathrm{Sc}{\left(H_{2}O\right)}_{1-9}^{3+}$ was replaced with a hydroxyl group to obtain the geometric configurations of mono- and di-hydroxy hydrates. Upon DFT optimization, it was observed that as the number of coordinated water ligands exceeded six, one of the $H_{2}O$ molecules in the initial $\mathrm{Sc}\left(\mathrm{OH}\right){\left(H_{2}O\right)}_{7}^{2+}$ structure detached; similarly, one of the $H_{2}O$ molecules in $\mathrm{Sc}{\left(\mathrm{OH}\right)}_{2}{\left(H_{2}O\right)}_{6}^{+}$ detached when the number of coordinated water molecules exceeded five. The most stable coordinated structures of the mono- and di-hydroxy hydrates are $\mathrm{Sc}\left(\mathrm{OH}\right){\left(H_{2}O\right)}_{6}^{2+}$ and $\mathrm{Sc}{\left(\mathrm{OH}\right)}_{2}{\left(H_{2}O\right)}_{5}^{+}$, respectively, as shown in Figure 3. As shown in Table 2, as the hydroxyl group replaces the coordinated water molecule, there is a slight increase in the length of the Sc-Ow bond, which increases slightly, and the binding energy decreases further. Thus, the binding energy of $\mathrm{Sc}{\left(\mathrm{OH}\right)}_{2}{\left(H_{2}O\right)}_{5}^{+}$ (−3661.44 KJ/mol) is lower than that of $\mathrm{Sc}\left(\mathrm{OH}\right){\left(H_{2}O\right)}_{6}^{2+}$ (−3180.48 KJ/mol) and $\mathrm{Sc}{\left(H_{2}O\right)}_{8}^{3+}$ (−2629.44 KJ/mol). Therefore, it is inferred that $\mathrm{Sc}{\left(\mathrm{OH}\right)}_{2}{\left(H_{2}O\right)}_{5}^{+}$ is the most stable structure of the Sc hydrate, and this structure is used for further calculations and analyses of its adsorption on the surface of kaolinite (001).

### 3.2. Outer Layer Adsorption of $Sc{\left(OH\right)}_{2}{\left(H_{2}O\right)}_{5}^{+}$ on the (001)Al-OH Surface

**Figure 4:**
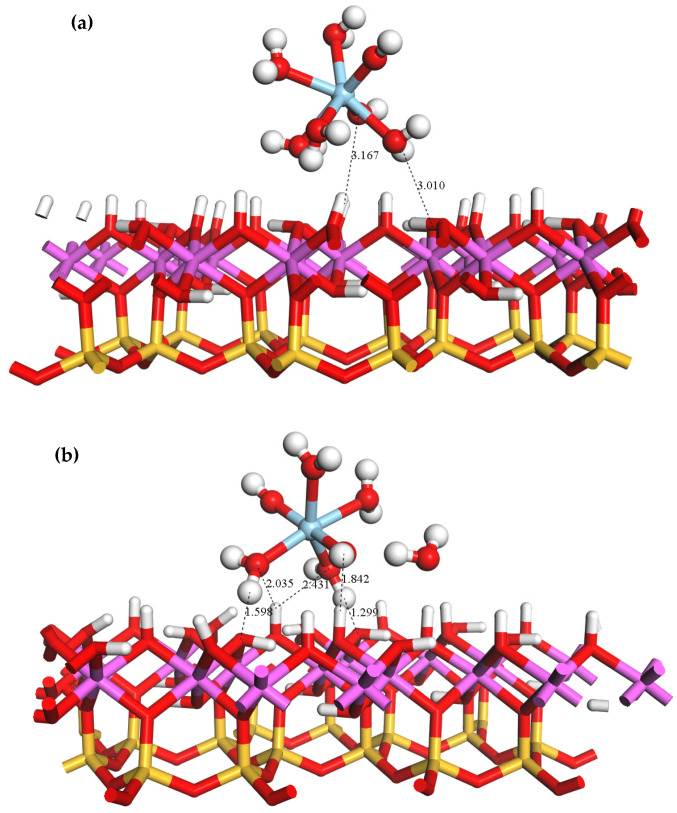
Equilibrium geometry of $\mathrm{Sc}{\left(\mathrm{OH}\right)}_{2}{\left(H_{2}O\right)}_{5}^{+}$ adsorbed on the outer layer of the (001)Al-OH surface (**a**) before adsorption; (**b**) after adsorption.

The equilibrium adsorption geometry of $\mathrm{Sc}{\left(\mathrm{OH}\right)}_{2}{\left(H_{2}O\right)}_{5}^{+}$ on the outer layer of the kaolinite (001)Al-OH surface is depicted in Figure 4. As shown, the Al-OH surface contains a large number of hydroxyl groups with a high steric hindrance, and one of the coordinated water molecules in the Sc hydrate was squeezed out upon its approach to the surface. Hydrogen bonds are established between the water molecules in $\mathrm{Sc}{\left(\mathrm{OH}\right)}_{2}{\left(H_{2}O\right)}_{5}^{+}$ and the hydroxyl groups on the surface of kaolinite. DFT optimizations resulted in a total of five hydrogen bonds between the adsorbate and adsorbent with bond lengths of 1.299, 1.598, 1.842, 2.035, and 2.431 Å. The equilibrium geometrical parameters and adsorption energies of $\mathrm{Sc}{\left(\mathrm{OH}\right)}_{2}{\left(H_{2}O\right)}_{5}^{+}$ bound to the Al-OH surface are provided in Table 3. Following the adsorption of $\mathrm{Sc}{\left(\mathrm{OH}\right)}_{2}{\left(H_{2}O\right)}_{5}^{+}$, the average Sc-Ow bond length decreased from 2.22 to 2.14 Å. This reduction can be attributed to the compression of water ligands near the surface and the detachment of one of the coordinated water molecules, resulting in a tighter bond between ${\mathrm{Sc}}^{3+}$ and the surrounding water ligands. The adsorption energy of $\mathrm{Sc}{\left(\mathrm{OH}\right)}_{2}{\left(H_{2}O\right)}_{5}^{+}$ is −522.24 KJ/mol.

**Figure 5:**
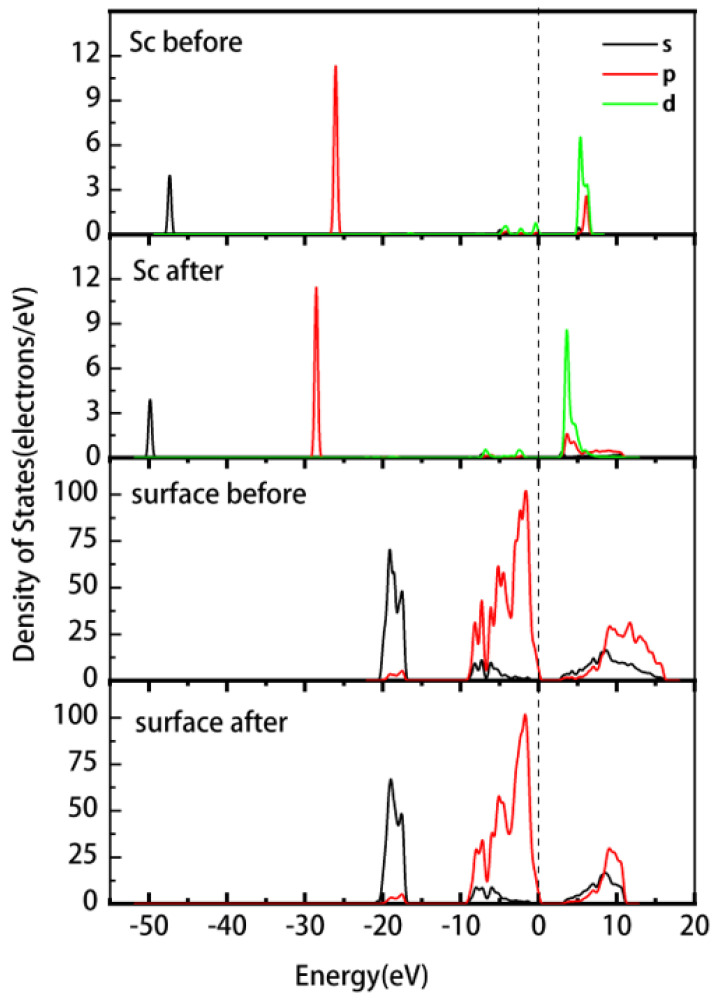
PDOS of Sc and the (001) Al-OH surface before and after the outer layer adsorption of the Sc hydrate.

Figure 5 shows the PDOS of the Sc ion and kaolinite surface before and after the adsorption of $\mathrm{Sc}{\left(\mathrm{OH}\right)}_{2}{\left(H_{2}O\right)}_{5}^{+}$ on the outer layer of the (001)Al-OH surface. The PDOS of the Sc ion is overall shifted towards lower energy after adsorption. The non-localization of 3d orbitals above the Fermi energy level is enhanced, and the density of states peak shifts from 4.6–6.9 eV to 2.6–7.5 eV. The 3p orbitals change from −(26.7–25.3) eV to −(29.2–27.8) eV. The Al-OH surface has a slight shift in the density of states towards lower energies after adsorption, and the 2p orbital near the Fermi energy level changes from −9.6–0.74 eV to −9.9–0.75 eV. The system becomes more stable, and there is a significant enhancement in the localization of the 2s and 2p orbitals in the conduction band.

The Mulliken atomic population analysis of the Sc hydrate before and after its adsorption is presented in Table 4. The data indicate that, upon adsorption, the 3s and 3p orbitals of Sc gain 0.02e and 0.04e, whereas the 3d orbital loses 0.11e. Due to the presence of hydrogen bonding interactions rather than coordination bonds, the total charge change is minimal (−0.05e), which is also consistent with the subtle changes observed in the PDOS plot. This indicates that the charge transfer from $\mathrm{Sc}{\left(\mathrm{OH}\right)}_{2}{\left(H_{2}O\right)}_{5}^{+}$ to the Al-OH surface is small.

### 3.3. Adsorption of $Sc{\left(OH\right)}_{2}{\left(H_{2}O\right)}_{5}^{+}$ on the Outer Layer of the (00−1)Si-O Surface

**Figure 6:**
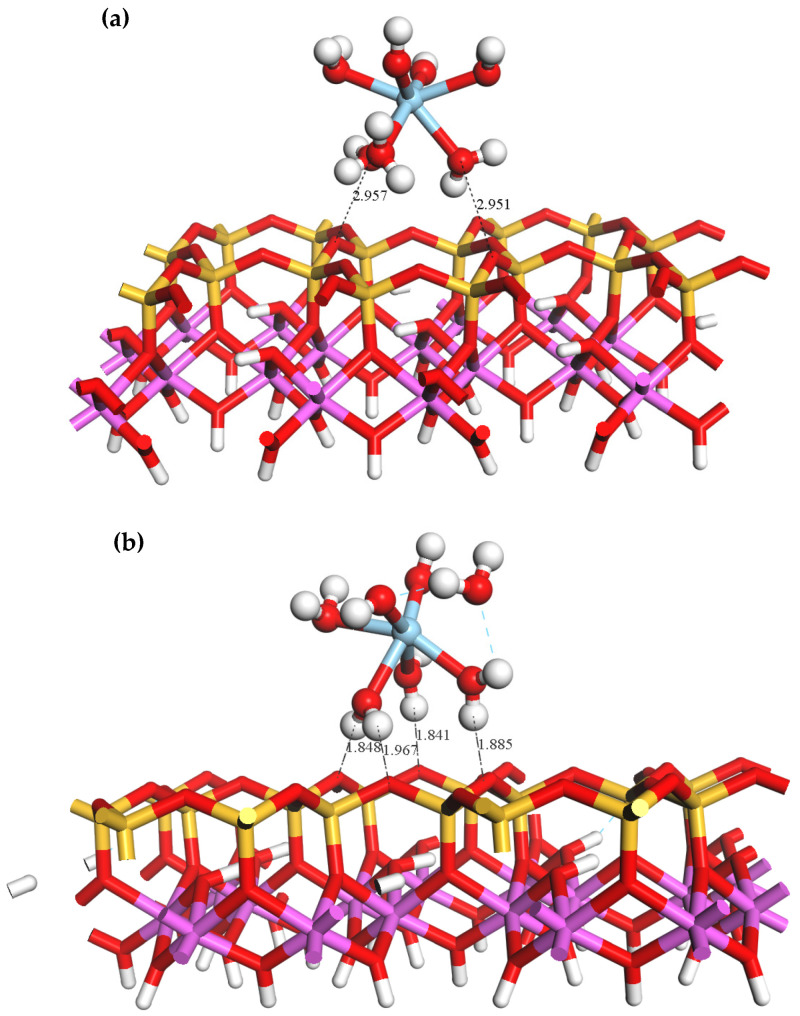
Equilibrium geometry for the adsorption of $\mathrm{Sc}{\left(\mathrm{OH}\right)}_{2}{\left(H_{2}O\right)}_{5}^{+}$ on the outer layer of the (00−1)Si-O surface (**a**) before adsorption; (**b**) after adsorption.

Figure 6 illustrates the adsorption configuration of $\mathrm{Sc}{\left(\mathrm{OH}\right)}_{2}{\left(H_{2}O\right)}_{5}^{+}$ on the outer layer of the kaolinite (00−1)Si-O surface. Near the (00−1)Si-O surface, one of the coordinated water molecules in $\mathrm{Sc}{\left(\mathrm{OH}\right)}_{2}{\left(H_{2}O\right)}_{5}^{+}$ detaches from the hydrate. This is in contrast to the (001)Al-OH surface, which does not have hydroxyl groups but is abundant in saturated oxygen atoms. The optimized surface oxygen atoms (O_s_) on the outer layer form four hydrogen bonds with the hydrogen atoms (H_w_) in the $H_{2}O$ ligands of the Sc hydrate and the H-bond lengths are 1.841, 1.848, 1.885, and 1.967 Å. The corresponding structural parameters and adsorption energies are presented in Table 5.

After adsorption occurs, the average Sc-O_w_ bond length experiences a slight reduction from 2.22 to 2.17 Å. This decrease can be attributed to the detachment of one coordinated water ligand and the steric hindrance present at the surface. The adsorption energy of $\mathrm{Sc}{\left(\mathrm{OH}\right)}_{2}{\left(H_{2}O\right)}_{5}^{+}$ on the Si-O surface is significantly lower (−648.96 KJ/mol) compared to the Al-OH surface (−522.24 KJ/mol). This is possibly due to the steric hindrance created by the hydroxyl groups on the Al-OH surface, which prevent $\mathrm{Sc}{\left(\mathrm{OH}\right)}_{2}{\left(H_{2}O\right)}_{5}^{+}$ from approaching the surface. Based on the analysis, it can be concluded that the outer layer adsorption of $\mathrm{Sc}{\left(\mathrm{OH}\right)}_{2}{\left(H_{2}O\right)}_{5}^{+}$ is more likely to occur on the Si-O surface of kaolinite.

**Figure 7:**
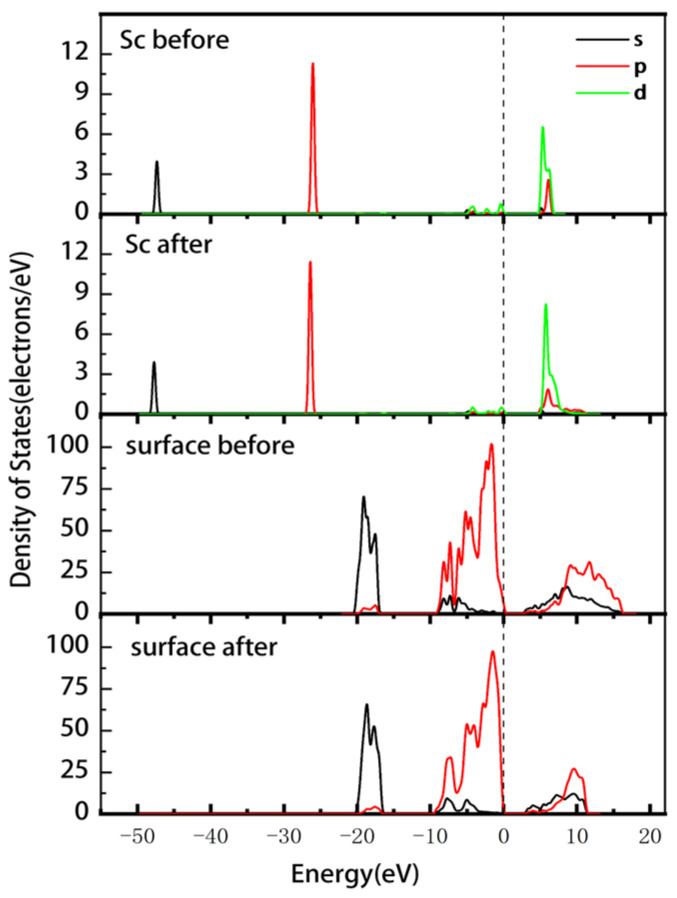
PDOS of Sc and the (00−1)Si-O surface before and after the outer layer adsorption of the Sc hydrate.

Figure 7 displays the PDOS of the Sc ion and the surface before and after adsorption. The change in Sc at the Fermi energy level was not significant; the energies of the 3d and 2p orbitals in the conduction band changed from 4.7–6.8 eV before adsorption to 4.4–11.6 eV after adsorption. Thus, the non-localization of the electrons was enhanced, and the overall shift toward lower energies was small. The Si-O surface has enhanced electron localization in the 2s and 2p orbitals in the conduction band. The energy of the 2p orbital at the Fermi level shifts from −8.6–0.5 eV to −9.6–0.25 eV, indicating a slight reduction in the reactivity of the Si-O surface. The Mulliken atomic population of Sc (Table 6) indicates that the 3s and 3p orbitals of Sc gain 0.02e each, the 3d orbitals lose 0.15e, and the total charge increases from 1.71e to 1.82e. The adsorption charge transfer of $\mathrm{Sc}{\left(\mathrm{OH}\right)}_{2}{\left(H_{2}O\right)}_{5}^{+}$ on the (00−1) Si-O surface is more significant compared to the data in Table 4. Therefore, it can be inferred that outer layer adsorption is more likely to occur on this surface.

### 3.4. Inner Layer Adsorption on the (001)Al-OH Surface

**Figure 8:**
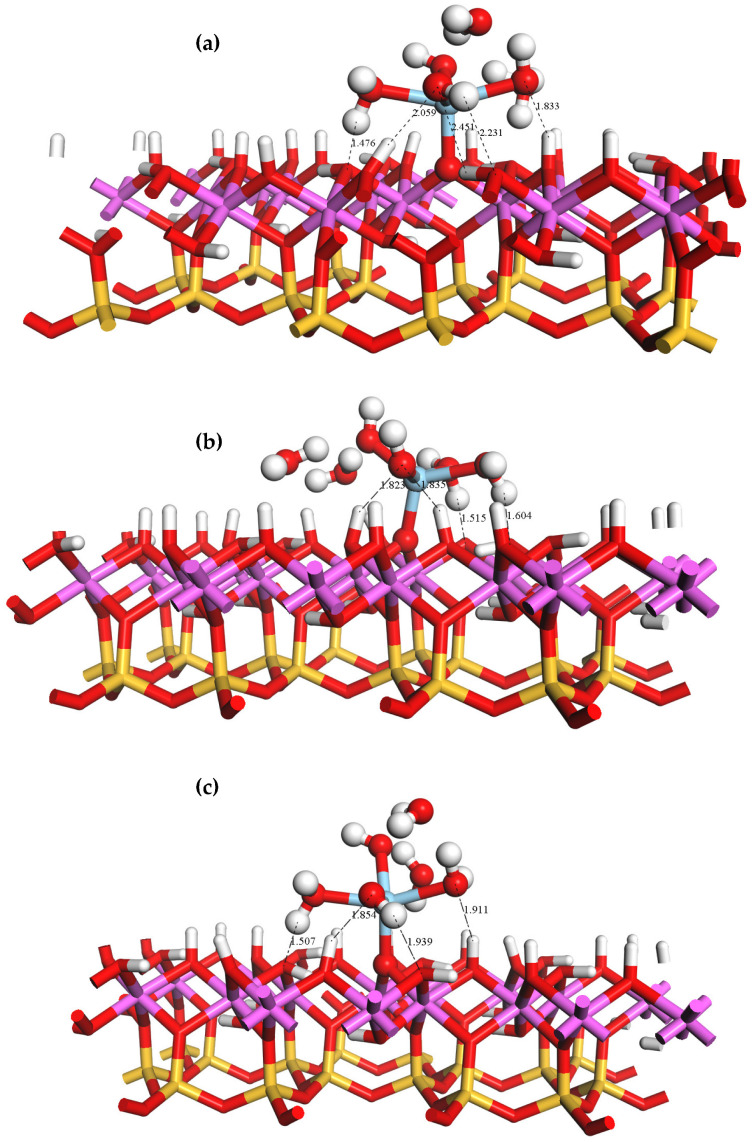
Equilibrium geometries of $\mathrm{Sc}{\left(\mathrm{OH}\right)}_{2}{\left(H_{2}O\right)}_{5}^{+}$ adsorbed on the inner layer O_u_ (**a**), O_l_ (**b**), and O_t_ (**c**) sites of the (001) Al-OH surface.

The adsorption behavior of rare earth ions on the surface of kaolinite in a liquid phase is pH-dependent. If the pH is higher than the point of zero charge of kaolinite, the surface hydroxyl groups lose their protons and become negatively charged. In such scenarios, the rare earth hydrate is bound to the deprotonated Al-OH surface through inner layer adsorption, specifically by forming coordination bonds with the oxygen atoms. Three forms of hydroxyl groups are present on the optimized Al-OH surface: tilted (OtH), lying (O_l_H), and upright (O_u_H) hydroxyl groups. The geometrical configurations of the inner layer adsorption forms of the Sc ions on these three deprotonated oxygen atoms are shown in Figure 8. When adsorbed in the inner layer, the Sc ion in $\mathrm{Sc}{\left(\mathrm{OH}\right)}_{2}{\left(H_{2}O\right)}_{5}^{+}$ forms a monodentate adsorption structure by coordinating with the deprotonated oxygen atom (Os) on the Al-OH surface. Due to the steric hindrance imposed by the kaolinite surface, two water ligands are displaced from the coordination sphere of Sc and released as free water molecules after adsorption in all three configurations, and the coordination number of Sc decreases.

The equilibrium geometrical parameters and adsorption energies of the three adsorption configurations of the Sc hydrate are presented in Table 7. Unlike the outer layer adsorption case, inner layer adsorption involves both hydrogen bonding and coordination bonding mechanisms. The coordination bond between the Sc ion and the surface oxygen atom (Os) has a shorter bond length compared to the average Sc-O_w_ bond length.

$\mathrm{Sc}{\left(\mathrm{OH}\right)}_{2}{\left(H_{2}O\right)}_{5}^{+}$ has the lowest adsorption energy on O_l_, indicating that O_l_ is the best adsorption site. Upon adsorption at the O_l_ site, one hydroxyl group and two coordinated water molecules in the rare earth hydrate near the kaolinite surface form four hydrogen bonds with the oxygen atom near the kaolinite surface. The bond lengths of these hydrogen bonds are 1.515, 1.604, 1.823, and 1.835 Å. The inner layer adsorption mode exhibits significantly lower adsorption energy compared to the outer layer adsorption mode, indicating that the formation of coordination bonds in the inner layer adsorption enhances the stability of Sc adsorption.

**Figure 9:**
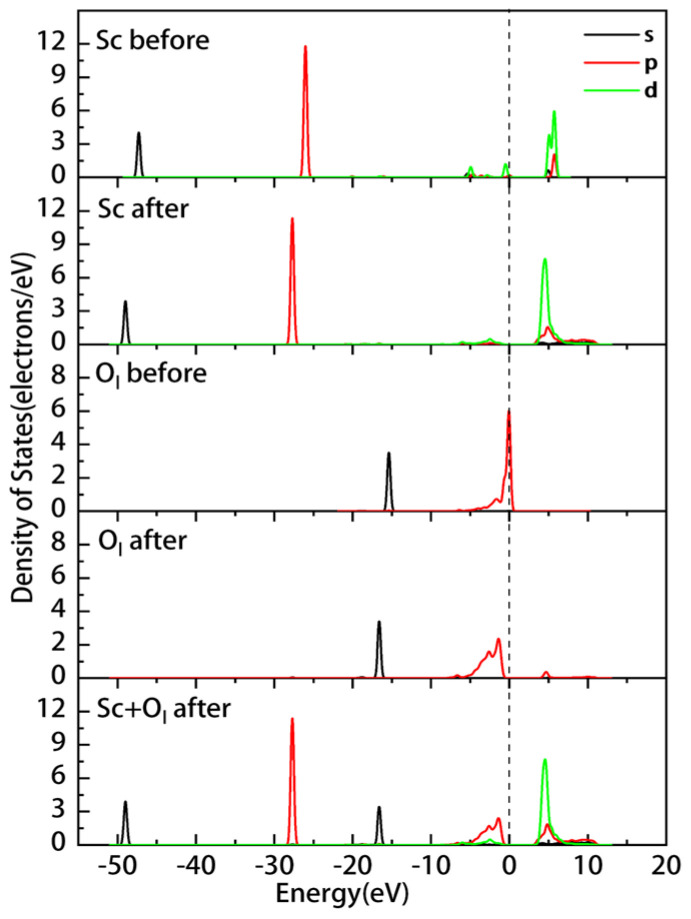
Partial density of states of Sc and Ol before and after the adsorption of the Sc hydrate on the inner layer.

Further, we analyzed the adsorption mechanism by calculating the PDOS and Mulliken atomic population of $\mathrm{Sc}{\left(\mathrm{OH}\right)}_{2}{\left(H_{2}O\right)}_{5}^{+}$ adsorbed at the O_l_ site. The results of this analysis are shown in Figure 9 and Table 8. The overall shift in the density of states peaks of Sc and surface oxygen O_l_ toward lower energies following the adsorption of the Sc hydrate indicates that the formation of coordination bonds between the adsorbed Sc and O_l_ results in a lower energy and more stable system. The 2p orbital of O_l_ moves away from the Fermi level after adsorption, resulting in a new peak at 4.6 eV. The overlapping of the 2p orbital of Ol and the 3d orbital of Sc in the energy range of −8 to −0.3 eV suggests the presence of bonding states in this region.

Analyses of the Mulliken charge of Sc and O_l_ before and after adsorption reveal that the Mulliken bond population value of the Sc-O bond is 0.48 and that the coordinative bonds formed on the surface are more covalent. After adsorption, the 4s orbital of Sc gains a charge of 0.02e, while the 3p and 3d orbitals lose charges of 0.13e and 0.18e, respectively, and the charge changes from 1.60 to 1.78 (i.e., 0.18 electrons are lost). Further, the 2s orbital of O_l_ loses 0.05e, the 2p orbital gains 0.16e, and the charge changes from −0.87 to −0.98 (0.11 electrons are gained). After the formation of the coordination bond, Sc transfers electrons to O_l_.

The outer layer adsorption of $\mathrm{Sc}{\left(\mathrm{OH}\right)}_{2}{\left(H_{2}O\right)}_{5}^{+}$ on the (001)Al-OH and (00−1)Si-O surfaces of kaolinite results in hydrogen bonding interactions. The adsorption energy of Sc on the outer layer of the (001)Al-OH surface is −522.24 KJ/mol, while on the Si-O surface, it is −648.96 KJ/mol. The steric hindrance of the hydroxyl groups on the (001)Al-OH surface prevents the rare earth ion from approaching. On the other hand, the oxygen atoms on the surface of (00−1)Si-O are strongly electronegative. Thus, $\mathrm{Sc}{\left(\mathrm{OH}\right)}_{2}{\left(H_{2}O\right)}_{5}^{+}$ is more stable when adsorbed on the outer layer of the (00−1)Si-O surface. At high pH values, the deprotonation of the hydroxyl group on the (001)Al-OH surface enables the formation of coordination bonds between the oxygen atom on the deprotonated surface (O_s_) and Sc ions, referred to as inner layer adsorption. The adsorption of $\mathrm{Sc}{\left(\mathrm{OH}\right)}_{2}{\left(H_{2}O\right)}_{5}^{+}$ on the deprotonated (001) surface results in three configurations, with the lowest adsorption energy (−653.32 KJ/mol) at the lying oxygen site (O_l_), which is significantly lower than the outer layer adsorption energy. Although the energy of the inner layer adsorption mode is lower, the energetic disadvantage of the surface deprotonation process and the fact that most of the rare earth species can be desorbed by ${\mathrm{NH}}_{4}^{+}$ ion exchange. Hence, the outer layer adsorption mode is the dominant mechanism for rare earth elements to bind with the kaolinite surface.

## 4. Conclusions

This study investigates the structures and bonding mechanisms of hydrated ${\mathrm{Sc}}^{3+}$,$\;\mathrm{Sc}{\left(\mathrm{OH}\right)}^{2+}$, and $\mathrm{Sc}{\left(\mathrm{OH}\right)}_{2\;}^{+}$ species adsorbed onto the surfaces of kaolinite (001)Al-OH and (00−1)Si-O using the plane-wave pseudopotential DFT method. Hydrated $\mathrm{Sc}{\left(\mathrm{OH}\right)}^{2+}$ was found to have a more stable structure and lower adsorption energy than hydrated ${\mathrm{Sc}}^{3+}$ and hydrated $\mathrm{Sc}{\left(\mathrm{OH}\right)}_{2}^{+}$ species. The best adsorption configuration for $\mathrm{Sc}{\left(\mathrm{OH}\right)}^{2+}$ in the liquid phase is $\mathrm{Sc}{\left(\mathrm{OH}\right)}_{2}{\left(H_{2}O\right)}_{5}^{+}$.

The adsorption energies of hydrated $\mathrm{Sc}{\left(\mathrm{OH}\right)}^{2+}$ on the outer layers of (001) and (00−1) surfaces of kaolinite are −522.24 and −648.96 KJ/mol, respectively. The adsorption process is primarily driven by hydrogen bonding interactions between the water ligands in the Sc hydrate and the surface of kaolinite.

Inner layer adsorption of hydrated $\mathrm{Sc}{\left(\mathrm{OH}\right)}^{2+}$ on the deprotonated hydroxyl group of the kaolinite (001) surface occurred in three adsorption configurations (O_u_, O_l_, and O_t_), with the lowest adsorption energy at the O_l_ site (−653.32 KJ/mol). In addition to hydrogen bonds, coordination bonding (Sc-O_S_) was also observed between Sc and oxygen atoms of the deprotonated hydroxyl groups on the surface, resulting in higher adsorption energy than that on the outer layer. According to the PDOS and Mulliken population analysis, the coupling between the 3d orbital of Sc and the 2p orbital of O_s_ to form a bonding state is the main contributor to the Sc-O_S_ coordination bond.

Although the inner layer adsorption exhibits lower adsorption energy compared to the outer layer adsorption, the presence of hydrated Sc ions primarily occurs in the outer layer adsorption mode on the kaolinite (001)Al-OH surface. The results indicate that inner layer adsorption exhibits greater stability compared to outer layer adsorption. However, the extent of deprotonation of the (001) surface in high-pH solutions is constrained by the energy required to deprotonate the inner layer hydroxyl groups. Most rare earth ions can be desorbed through ion exchange with ${\mathrm{NH}}_{4}^{+}$. It can therefore be concluded that the main adsorption mode for rare earth ions is on the outer layer of the kaolinite surface. To prevent the deprotonation of the hydroxyl groups on the (001) surface of kaolinite, a leaching process at a lower pH can be employed to facilitate the exchange of rare earth ions.

## Figures and Tables

**Table 1:** Geometric structural parameters and binding energies of the equilibrium structure of $\mathrm{Sc}{\left(H_{2}O\right)}_{1-9}^{3+}$.

n ^a^	$R{{\left(Sc-O_{w}\right)}_{min\;}}^{b}$ /Å	$R{\left(Sc-O_{w}\right)}_{max}$ /Å	$R{\left(Sc-O_{w}\right)}_{avg}$ /Å	E_binding_ /KJ·mol^−1^	Sc Charge /e
1	1.94309	1.94309	1.94309	−709.44	2.52
2	2.20016	2.20044	2.2003	−1208.64	2.3
3	2.03104	2.03233	2.03155	−1607.04	2.19
4	2.06466	2.07317	2.06876	−1935.36	2.13
5	2.08986	2.1409	2.11343	−2184	2.08
6	2.13676	2.14966	2.14483	−2404.8	2.07
7	2.17566	2.24127	2.20447	−2528.64	2.04
8	2.18095	2.35703	2.26569	−2629.44	2.03

^a^ Number of coordinated water molecules. ^b^ Distance of Sc from the oxygen atom of the coordinated water.

**Table 2:** Equilibrium geometrical parameters and binding energies of $\mathrm{Sc}\left(\mathrm{OH}\right){\left(H_{2}O\right)}_{6}^{2+}$ and $\mathrm{Sc}{\left(\mathrm{OH}\right)}_{2}{\left(H_{2}O\right)}_{5}^{+}$.

Sample	R(Sc-O_w_)_min_ /Å	R(Sc-O_w_)_max_ /Å	R(Sc-O_w_)_avg_ /Å	E_binding_ /KJ·mol^−1^	Charge on Sc/e
$\mathrm{Sc}\left(\mathrm{OH}\right){\left(H_{2}O\right)}_{6}^{2+}$	1.85728	2.31498	2.21025	−3180.48	1.77
$\mathrm{Sc}{\left(\mathrm{OH}\right)}_{2}{\left(H_{2}O\right)}_{5}^{+}$	2.34878	2.34878	2.22228	−3661.44	1.71

**Table 3:** Equilibrium geometrical parameters and adsorption energies for the adsorption structures of $\mathrm{Sc}{\left(\mathrm{OH}\right)}_{2}{\left(H_{2}O\right)}_{5}^{+}$ on the outer layer of the (001)Al-OH surface.

Name	State	N	R(Sc-O_w_) /Å	R(Sc-O_w_)_avg_ /Å	Eads /KJ·mol^−1^
$\mathrm{Sc}{\left(\mathrm{OH}\right)}_{2}{\left(H_{2}O\right)}_{5}^{+}$	Before	7	1.90, 1.98, 2.30, 2.31,2.34, 2.35, 2.38	2.22	−522.24
	After	6	1.90, 2.02, 2.12, 2.22,2.26, 2.32	2.14	

**Table 4:** Mulliken atomic population of Sc adsorbed on the outer layer of the (001) Al-OH surface.

State	3s	3p	3d	Total	Charge/e
Before adsorption	2.14	5.94	1.21	9.29	1.71
After adsorption	2.16	5.98	1.1	9.24	1.76
Δcharge ^a^	0.02	0.04	−0.11	−0.05	0.05

^a^ Amount of change in charge before and after adsorption.

**Table 5:** Equilibrium geometries and adsorption energies for the adsorption of $\mathrm{Sc}{\left(\mathrm{OH}\right)}_{2}{\left(H_{2}O\right)}_{5}^{+}$ on the outer layer of the (00−1)Si-O surface.

Name	State	N	R(Sc-O_w_) /Å	R(Sc-O_w_)_avg_ /Å	Eads /KJ·mol^−1^
$\mathrm{Sc}{\left(\mathrm{OH}\right)}_{2}{\left(H_{2}O\right)}_{5}^{+}$	Before	7	1.90, 1.98, 2.30, 2.31, 2.34, 2.35, 2.38	2.22	−648.96
	After	6	1.95, 1.99, 2.19, 2.23, 2.29, 2.34	2.17	

**Table 6:** Mulliken atomic population of Sc adsorbed on the outer layer of the (00−1) Si-O surface.

State	3s	3p	3d	Total	Charge/e
Before adsorption	2.14	5.94	1.21	9.29	1.71
After adsorption	2.16	5.96	1.06	9.18	1.82
Δcharge	0.02	0.02	−0.15	−0.11	0.11

**Table 7:** Equilibrium geometries and adsorption energies of three configurations of Sc adsorbed on the inner layer of the (001)Al-OH surface.

Location	N	R(Sc-O_w_)_avg_ /Å	R(Sc-O_s_) ^a^ /Å	Eads /KJ·mol^−1^
O_u_	4	2.13	1.95	−643.68
O_l_	4	2.13	1.94	−653.32
O_t_	4	2.17	2.00	−595.68

^a^ Distance between Sc and the surface deprotonated oxygen.

**Table 8:** Mulliken atomic populations of Sc and O_l_ before and after adsorption, along with the Mulliken bond population of the Sc-O_l_ bond.

State	Sc					O_l_				Sc-O_l_ ^a^
	s	p	d	Total	Charge	s	p	Total	Charge	
Before	2.14	6.01	1.25	9.40	1.60	1.91	4.96	6.87	−0.87	0.48
After	2.16	5.94	1.12	9.22	1.78	1.86	5.12	6.98	−0.98	
Δcharge	0.02	−0.07	−0.13	−0.18	0.18	−0.05	0.16	0.11	−0.11	

^a^ Bond population value for coordination bond formed by Sc and surface oxygen.

## Data Availability

The data presented in this study are available upon request from the corresponding authors.
